# Genomic Insights into Vietnamese Extended-Spectrum β-Lactamase-9-Producing Extensively Drug-Resistant *Pseudomonas aeruginosa* Isolates Belonging to the High-Risk Clone ST357 Obtained from Bulgarian Intensive Care Unit Patients

**DOI:** 10.3390/pathogens13090719

**Published:** 2024-08-25

**Authors:** Tanya Strateva, Alexander Stratev, Slavil Peykov

**Affiliations:** 1 Department of Medical Microbiology “Corr. Mem. Prof. Ivan Mitov, MD, DMSc”, Faculty of Medicine, Medical University of Sofia, 2 Zdrave Str., 1431 Sofia, Bulgaria; spejkov@biofac.uni-sofia.bg; 2 Intensive Care Unit, University Multiprofile Hospital for Active Treatment ‘St. Ivan Rilski’, 15 Acad. Ivan Geshov Blvd., 1431 Sofia, Bulgaria; astratev@gmail.com; 3 Department of Anaesthesiology and Intensive Care, Faculty of Medicine, Medical University of Sofia, 1 St. Georgi Sofiyski Str., 1431 Sofia, Bulgaria; 4 Department of Genetics, Faculty of Biology, University of Sofia ‘St. Kliment Ohridski’, 8 Dragan Tzankov Blvd., 1164 Sofia, Bulgaria; 5 BioInfoTech Laboratory, Sofia Tech Park, 111 Tsarigradsko Shose Blvd., 1784 Sofia, Bulgaria

**Keywords:** *Pseudomonas aeruginosa*, carbapenem resistance, extensive drug resistance, VEB-9 extended-spectrum β-lactamase, high-risk clone ST357, exotoxin U, whole-genome sequencing, phylogenomic analysis

## Abstract

Extensively drug-resistant *P. aeruginosa* (XDR-PA) has been highlighted as a serious public health threat. The present study aimed to explore the genomic characteristics of two Vietnamese extended-spectrum β-lactamase-9 (VEB-9)-producing XDR-PA isolates from Bulgaria in comparison to all *bla*_VEB-9_-positive strains with available genomes. The isolates designated Pae51 and Pae52 were obtained from tracheobronchial aspirates of intensive care unit (ICU) patients. Antimicrobial susceptibility testing, whole-genome sequencing, RT-qPCR, and phylogenomic analysis were performed. Pae51 and Pae52 were resistant to most antipseudomonal β-lactams including carbapenems, aminoglycosides, and fluoroquinolones but remained susceptible to colistin and cefiderocol. Numerous resistance determinants were detected: *bla*_VEB-9_, *bla*_PDC-3_, *bla*_OXA-10_, *bla*_OXA-50_, *aac(6′)-II*, *ant(2″)-Ia*, *ant(3″)-IIa*, *aph(3′)-IIb*, *cprP*, *catB7*, *dfrB2*, *sul1*, *fosA*, and *tet(A)*. Both isolates carried complex integrons with *bla*_VEB-9_ and *tet(A)* embedded next to the conservative 3′ end sequences. A variety of virulence factors were also identified, including the type III secretion system exotoxin U. Pae51 and Pae52 differed by only four SNPs and belonged to the high-risk clone ST357. To our knowledge, this is the first report of *bla*_VEB-9_-positive XDR-PA isolates in Bulgaria presenting a detailed genomic analysis. The development of novel antimicrobial strategies for such pathogens should be an essential part of infection control stewardship practices in ICU wards.

## 1. Introduction

*Pseudomonas aeruginosa*, a ubiquitous Gram-negative bacterium considered one of the paradigms of antimicrobial resistance (AMR), is among the main causes of hospital-acquired and chronic, hard-to-eradicate infections associated with high healthcare costs, as well as significant morbidity and mortality rates [1,2]. It is one of the six leading mortality-causing pathogens, which were collectively accountable for 929,000 deaths attributable to AMR and 3.57 million deaths associated with AMR in 2019 worldwide [3]. Predisposing factors for infections due to *P. aeruginosa* include mechanical ventilation, catheterization, burn victims, innate or acquired immunodeficiencies, immunosuppressive therapy, cystic fibrosis, and metabolic disorders [4,5,6,7,8,9]. *P. aeruginosa* is mainly considered an opportunistic, nosocomial pathogen, which is commonly found in intensive care units (ICUs) and surgical settings, where the extensive use of antimicrobials has allowed for the selection of these microorganisms [10]. During the global pandemic, it established itself as one of the most common pathogens causing respiratory co-infections and bacteremia in critically ill COVID-19 patients [11,12].

The treatment of hospital-acquired infections (HAIs) caused by *P. aeruginosa* is challenging because of the organism’s intrinsic resistance to multiple antibiotics and its remarkable ability to acquire practical resistance to all relevant antipseudomonal antibiotics [13]. Therefore, carbapenem-resistant *P. aeruginosa* (CRPA) has been highlighted by the World Health Organization (WHO) as a major threat with critical priority for the development of new antibiotics [14]. This growing threat results from its extraordinary capacity to develop AMR via chromosomal mutations and from the increasing prevalence of transferable AMR determinants, particularly those encoding carbapenem-hydrolyzing enzymes and extended-spectrum β-lactamases (ESBLs) [15,16]. Worryingly, these mechanisms are often present simultaneously and confer complex resistant phenotypes. The European Centre for Disease Prevention and Control (ECDC) and U.S. Centers for Disease Control and Prevention classify such therapeutically problematic isolates of *P. aeruginosa* as multidrug-resistant (MDR-PA), extensively drug-resistant (XDR-PA), and pandrug-resistant (PDR-PA), while the Infectious Diseases Society of America/National Institutes of Health (IDSA/NIH) defines them as isolates with difficult-to-treat resistance (DTR-PA) [17,18].

Several surveys have provided evidence for the existence of ten XDR/DTR international *P. aeruginosa* high-risk clonal lineages, which have been successfully adapted to hospital settings worldwide [19,20]. Some of these, sequence types ST235, ST357, ST308, and ST298, include strains characterized by both problematic AMR and a highly virulent phenotype associated with a high mortality rate, likely due to the production of the ExoU cytotoxin [21]. In severe *P. aeruginosa* infections, the phospholipase activity of the type III secretion system (T3SS) ExoU effector protein induces lysis of target host cells and results in the poorest clinical outcomes [22,23].

Beyond molecular epidemiology analysis and a phenotypic assessment of resistance and virulence mechanisms, whole-genome sequencing (WGS) studies are generating relevant information to elucidate the evolving resistome/virulome of *P. aeruginosa* high-risk clones [24,25,26]. The application of WGS to AMR monitoring provides a deep understanding of the genetic mechanisms involved and easy identification of novel variants of ESBLs and metallo-β-lactamases (MBLs), which are most often the focus of current studies. On the other hand, high-resolution subtyping, comparing the genomes of *P. aeruginosa* strains of different geographic origins with identical genetic AMR determinants, is a powerful tool for discovering their evolutionary and epidemiological relationships.

Vietnamese extended-spectrum β-lactamase (VEB)-type enzymes of molecular class A and functional group 2be are among the most common β-lactamases identified in clinical *P. aeruginosa* isolates [20]. Their hydrolytic profile includes carboxypenicillins, ureidopenicillins, third and fourth generation cephalosporins, and aztreonam [27]. The in vitro activity of VEB-type ESBLs is well inhibited by clavulanate, but inhibition by avibactam is variable [28]. The detection of VEB ESBLs in *P. aeruginosa* strains began in the late 1990s and continues today with the identification of novel variants of the first enzyme in this group, VEB-1.

The present study aimed to explore the genomic characteristics of two VEB-9-producing XDR-PA isolates from ICU patients treated at a Bulgarian university hospital in comparison to all *bla*_VEB-9_-positive *P. aeruginosa* strains with available genomes.

## 2. Materials and Methods

### 2.1. Bacterial Strains and Clinical Case Presentation

Two XDR-PA respiratory isolates carrying determinants of a VEB-type ESBL variant previously undescribed in Bulgaria were identified during a WGS project regarding the resistome of nosocomial CRPA isolates. They were obtained from critically ill patients with ventilator-associated pneumonia (VAP) admitted to the University Hospital “St. Ivan Rilski” (405 beds). The hospital is a tertiary care hospital, located in the capital of Bulgaria, Sofia.

The first *P. aeruginosa* Pae51 isolate (designated Pae51) was obtained at the beginning of February 2020 from a tracheobronchial aspirate of a 26-year-old male with an intramedullary lymphoma tumor of the cervical spine. The patient had been admitted to the Clinic of Neurosurgery, where he underwent surgical decompression (laminectomy at the C_4_–C_6_) and tumor removal, several days prior to the isolation of Pae51. After the surgical procedure, he was transferred to the ICU with stable vital signs, spontaneous breathing via an endotracheal tube and persisting pre-operative neurological deficit in the upper and lower extremities. The early postoperative period was complicated by a developing weakness in the respiratory muscles and the onset of hypercapnia and hypoxemia, which required the initiation of controlled mechanical ventilation with positive end-expiratory pressure. During the next two weeks, the patient’s condition did not improve, and he developed pneumonia and septicemia. The blood culture taken was positive for *Enterococcus faecalis*. It was then decided that the patient should be moved to a specialized hospital in order to treat the respiratory failure.

The second *P. aeruginosa* Pae52 isolate (designated Pae52) was obtained at the end of April 2020 also from the tracheobronchial aspirate of a 68-year-old male with acute subarachnoid hemorrhage. The patient underwent emergency endovascular embolization of an aneurysm defect to the anterior communicating artery, several weeks prior to the isolation of Pae52. After the procedure, he was admitted to the ICU with signs of a newly developed ischemic lesion in the left hemisphere. He was then placed in a medically induced coma with thiopental and controlled mechanical ventilation was initiated. During the prolonged stay in the ICU, the patient never regained consciousness or spontaneous breathing. His condition deteriorated and became critical, manifesting with signs of pneumonia, septic shock, and worsening multiple organ failure. Despite the treatment, nearly two months after his hospitalization, the patient died from his illness.

*P. aeruginosa* ATCC 27,853 was used as a control strain for species identification and antimicrobial susceptibility testing.

### 2.2. Species Identification

Species identification was carried out using the BD Phoenix M50 automated system (BD, Franklin Lakes, NJ, USA). The identification of Pae51 and Pae52 was confirmed by analyzing the assembled draft genome sequence using the Microbial Genomes Atlas (MiGA) Web server [29]. The included workflow for the National Center for Biotechnology Information (NCBI) Genome Database, prokaryotic section, was followed with default settings.

### 2.3. Antimicrobial Susceptibility Testing

The antimicrobial susceptibility testing (AST) of the VEB-9-producing *P. aeruginosa* isolates was performed using the minimal inhibitory concentration (MIC) gradient method (MIC Test Strip; Liofilchem, Roseto degli Abruzzi, Italy) or the broth microdilution (BMD) method (ComASP Cefiderocol and ComASP Colistin, Liofilchem), according to the European Committee on Antimicrobial Susceptibility Testing (EUCAST) version 14.0, 2024 guidelines, with the following antimicrobial agents: piperacillin-tazobactam (TZP), ceftazidime (CAZ), cefepime (FEP), cefiderocol (CFDC), imipenem (IMP), meropenem (MEM), aztreonam (ATM), amikacin (AMK), tobramycin (TOB), ciprofloxacin (CIP), levofloxacin (LVX), and colistin (COL) [30]. MICs of several additional antibiotics, such as ceftazidime-avibactam (CZA), ceftolozane-tazobactam (CTT), imipenem-relebactam (IMR), and meropenem-vaborbactam (MEV), were determined using the Sensititre Gram Negative MDRGNXXF AST Plates (Thermo Fisher Scientific Inc., Waltham, MA, USA) following the manufacturer’s protocol.

### 2.4. Definitions of MDR-PA, XDR-PA, PDR-PA, and DTR-PA Isolates

According to the international expert proposal [17], MDR-PA isolates are non-susceptible to at least one agent in three or more categories, whereas XDR-PA isolates are non-susceptible to at least one agent in all but two or fewer categories, including aminoglycosides (AMK, TOB), antipseudomonal carbapenems (IMP, MEM), antipseudomonal cephalosporins (CAZ, FEP), antipseudomonal fluoroquinolones (CIP, LVX), antipseudomonal penicillins + β-lactamase inhibitors (TZP, ticarcillin-clavulanic acid), monobactams (ATM), and polymyxins (COL, polymyxin B). PDR-PA isolates are non-susceptible to all antimicrobial agents listed.

DTP-PA is defined by non-susceptibility to all of the following antimicrobials: CAZ, FEP, TZP, IMP, MEM, CIP, LVX, and ATM [18].

### 2.5. DNA Isolation

Total DNA from the investigated strains was isolated using the DNeasy Blood and Tissue Kit (QIAGEN, Hilden, Germany), according to the manufacturer’s instructions, from 3 mL of overnight cultures inoculated with a single colony.

### 2.6. Whole-Genome Sequencing (WGS)

The two *bla*_VEB-9_-positive *P. aeruginosa* isolates underwent WGS to facilitate comprehensive analyses of their resistomes and virulomes. The extracted genomic DNA was randomly fragmented, size-selected, ligated to adapters, and then PCR-amplified. Following these steps, the generated libraries were sequenced on an Illumina NovaSeq 6000 platform (Novogene, Cambridge, UK), producing 2 × 150 bp paired-end reads.

### 2.7. Draft Genome Assembly

The entire procedure encompassed several steps: quality control (FastQC v0.11.9, https://www.bioinformatics.babraham.ac.uk/projects/fastqc/, accessed on 15 July 2024), raw read preprocessing (Trimmomatic v0.38) [31], genome assembly (SPAdes v3.12.0) [32], and draft genome metrics evaluation (Quast v5.2.0) [33]. All software tools applied were part of the Galaxy online platform [34] and were operated with default parameters unless otherwise specified.

### 2.8. Resistome Analysis

Both assembled draft genome sequences were screened for AMR genes using ABRicate (v1.0.1) with the following settings: the Comprehensive Antibiotic Resistance Database (CARD) [35], minimum DNA identity (70%), and minimum DNA coverage (60%).

Additionally, the *gyrA* and *parC* coding sequences were manually analyzed for mutations in their quinolone resistance-determining regions (QRDRs). Finally, the full lengths of *oprD* and *mexT* were screened for carbapenem resistance-related missense, frameshift, and indel variants. Both analyses were performed using blastn comparisons with the corresponding sequences in the *P. aeruginosa* PAO1 strain (Gene IDs: 881970, 880417).

### 2.9. Reverse Transcription Quantitative Real-Time Polymerase Chain Reaction (RT-qPCR)

The mRNA levels of the *oprD* gene, encoding the outer membrane porin OprD, were measured by two-step RT-qPCR as previously described [36]. The imipenem-susceptible *P. aeruginosa* PAO1 strain was used as a calibrator for the evaluation step. In brief, DNase-treated RNA isolated from cultures in the log phase was subjected to reverse transcription using the SuperScript™ III First-Strand Synthesis System (Thermo Fisher Scientific, Carlsbad, CA, USA) with random hexamers. Concurrently, a control reverse transcription reaction without the enzyme was performed to exclude the possibility of genomic DNA contamination. The resulting cDNAs were appropriately diluted, and 2 μL of each dilution was combined with 8 μL of primer mix [36] and 10 μL of iTaq Universal SYBR Green Supermix (Bio-Rad Laboratories, Hercules, CA, USA) to prepare the qPCR reactions. They were conducted using a Rotor-Gene Q real-time PCR System (QIAGEN). The *oprD* mRNA expression was calculated using the 2^−ΔΔCt^ method [37] with an expression rate of ≤30% compared to the *P. aeruginosa* PAO1 strain considered indicative of diminished gene expression.

### 2.10. Virulome Analysis

The assembled genomes of both isolates were screened for virulence determinants using the VFanalyzer tool available at the Virulence Factor Database (VFDB) [38] with default settings.

### 2.11. Multilocus Sequence Typing (MLST) and O-Antigen Serotyping Analyses

The MLST analysis was conducted on the assembled contigs using the MLST tool (Galaxy Version 2.19.0, https://usegalaxy.eu/). The utilized *P. aeruginosa* MLST scheme incorporates internal fragments of the following seven housekeeping genes: *acsA* (acetyl coenzyme A synthetase), *aroE* (shikimate dehydrogenase), *guaA* (GMP synthase), *mutL* (DNA mismatch repair protein), *nuoD* (NADH dehydrogenase I chain C, D), *ppsA* (phosphoenolpyruvate synthase), and *trpE* (anthralite synthetase component I) [39].

The Pae51 and Pae52 isolates were assigned O-serotypes in silico using the Pseudomonas aeruginosa serotyper (PAst) tool v1.0 [40].

### 2.12. Phylogenomic Analysis

The phylogenomic analysis included our VEB-9-producing XDR-PA isolates (*n* = 2) and all *bla*_VEB-9_-positive *P. aeruginosa* strains with genomes deposited in the NCBI Nucleotide database (*n* = 20) and The Pseudomonas Genome Database [41] (*n* = 116) as of 1 July 2024 (search keywords: *Pseudomonas aeruginosa*, VEB-9). All sequences from both sources underwent quality evaluation (Quast v5.2.0) [33], and draft genomes with a total number of contigs (>1000 bp) greater than 200 and/or an L50 value greater than 25 were excluded from the subsequent analysis. The remaining nonduplicate genomes (*n* = 76) were subjected to MLST analysis via the above-mentioned MLST tool and screening for AMR genes (ABRicate v1.0.1). After confirming their VEB-9 positivity, all sequences were annotated using Prokka v1.14.6 [42]. Subsequently, the pangenome pipeline Roary v3.13.0 [43] was employed to generate a core gene alignment with a minimum blastp identity of 95% and a core definition threshold of 99%. A phylogenetic tree was then constructed using the RAxML program v8.2.12, employing the neighbor-joining clustering method with 1000 bootstraps [44]. The Interactive Tree Of Life portal was utilized to create a graphical representation of the phylogenetic tree [45].

## 3. Results

### 3.1. Antimicrobial Susceptibility

The AST profiles of the two *P. aeruginosa* isolates studied are presented in Table 1. As shown, Pae51 and Pae52 were resistant to most antipseudomonal β-lactams including carbapenems (MIC values of IMP and MEM > 32 mg/L), aminoglycosides, and fluoroquinolones, but remained susceptible to colistin (1 mg/L) and cefiderocol (0.5–1 mg/L). Both isolates were classified as XDR-PA, as well as DTR-PA.

### 3.2. Draft Genome Assemblies: Evaluation and Comparison

The two assembled draft genomes showed an identical size of 6.72 Mb, and their GC% content was approximately 66% (Table 2). These values are comparable with the accessible data from sequenced *P. aeruginosa* genomes. Also, Pae51 and Pae52 belonged to the epidemic high-risk ST357, which is the founder of the CC357 clonal complex, and the *P. aeruginosa* O11 serotype.

### 3.3. WGS-Based Resistome Analysis

**Figure 1:**
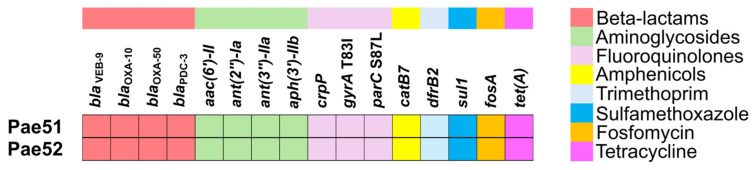
Resistome analysis of two *bla*_VEB-9_-positive *P. aeruginosa* isolates obtained from critically ill patients admitted to the University Hospital “St. Ivan Rilski”, Bulgaria.

Resistome analysis revealed the existence of *bla*_VEB-9_, encoding the VEB-9 ESBL, in both isolates studied. In addition, Pae51 and Pae52 possessed genes for the class C AmpC β-lactamase known as Pseudomonas-Derived Cephalosporinase (*bla*_PDC-3_), two genes for class D β-lactamases (*bla*_OXA-10_ and *bla*_OXA-50_), several determinants encoding aminoglycoside-modifying enzymes, *crpP* (a CrpP enzyme able to phosphorylate ciprofloxacin), *catB7* (chloramphenicol acetyltransferase B7), *dfrB2* (dihydrofolate reductase B2), *sul1* (dihydropteroate synthase type-1), *fosA* (Mn^2+^- and K^+^-dependent glutathione S-transferase associated with fosfomycin resistance), and *tet(A)* (Tet(A) efflux pump). All identified AMR genes in the two sequenced genomes are illustrated in Figure 1. A comparison of the QRDRs of Pae51 and Pae52 with the corresponding region of *P. aeruginosa* PAO1 revealed that the investigated isolates possessed two amino acid substitutions (p.T83I in *gyrA* and p.S87L in *parC*) with a role in fluoroquinolone resistance.

**Figure 2:**
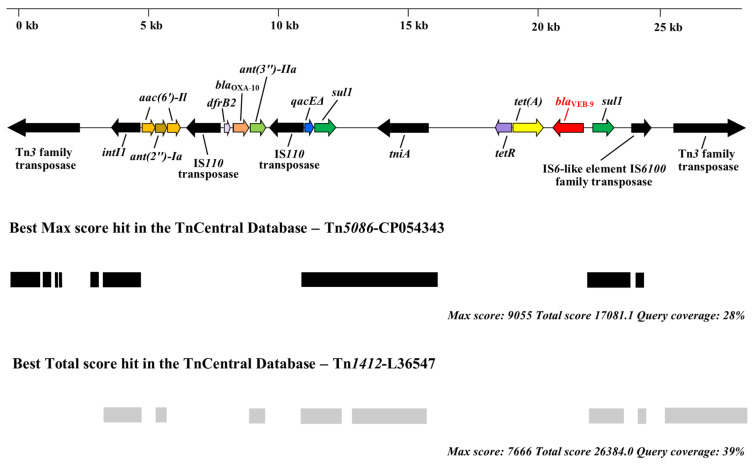
Schematic representation of the *bla*_VEB-9_ environment in the *P. aeruginosa* Pae51 and Pae52 isolates (ST357) from Bulgaria. The figure also shows the presence of genes associated with mobile genetic elements and the best matches for the VEB-9 region in the TnCentral database.

Our manual analysis of the genetic environment of the *bla*_VEB-9_ gene in the assembled genomes of Pae51 and Pae52 revealed characteristic features indicative of a class 1 integron with a complex structure (Figure 2). The *bla*_VEB-9_ and *tet*(A) genes were located near its 3′ end, which is indicated by the presence of *sul1*. Various genes related to mobile genetic elements are present upstream and downstream (Figure 2, marked in black). A comparative BLASTn analysis of the entire sequence in the TnCentral database [46] revealed various hits, with Tn*5086*-CP054343 and Tn*1412*-L36547 showing the highest max and total scores, respectively.

The size and sequencing coverage of the corresponding contigs suggest that the *bla*_VEB-9_ genes, carried by the *P. aeruginosa* Pae51 and Pae52 isolates, are located on their chromosomes.

**Figure 3:**
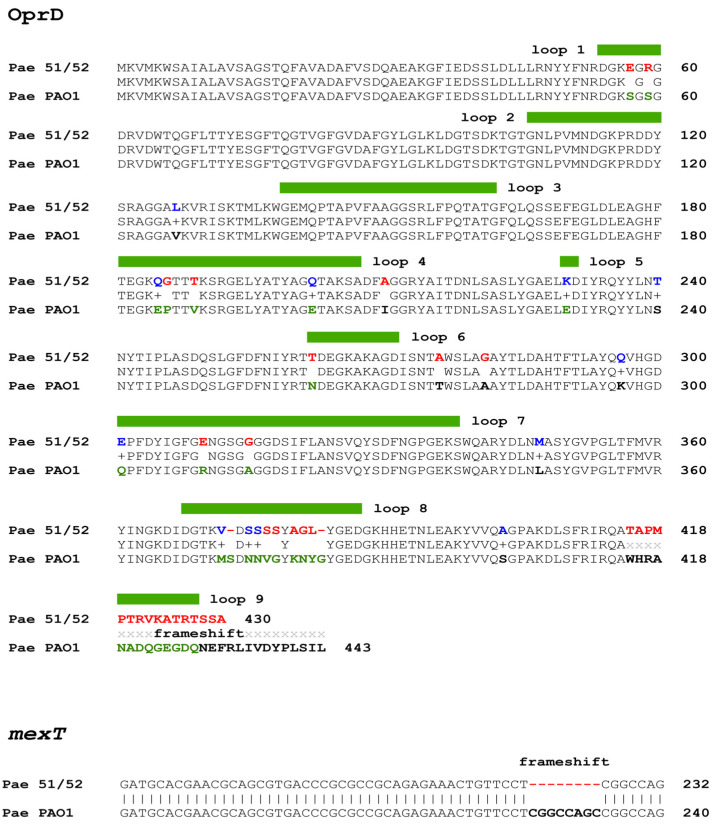
Amino acid sequences of the Pae51/Pae52 OprD sequence aligned with that of the *P. aeruginosa* PAO1 strain. Color coding in the Pae51/52 line represents similar (conservative—given in blue) and dissimilar (non-conservative—given in red) substitutions, determined by the BLASTP alignment/BLOSUM62 matrix. The loop regions, as determined by the crystal structure of the porin protein, are demarcated with a thick green line over the alignment. Mutations inside loops are marked in green (PAO1 line).

The coding sequence of the *oprD* gene was screened for mutations, revealing that both Pae51 and Pae52 harbor identical sequence variants. All identified missense and indel variants, compared to the corresponding region in *P. aeruginosa* PAO1, are shown in Figure 3. Additionally, the OprD porin produced by Pae51 and Pae52 was compared to the Non-redundant Protein Sequence (nr) database via BLASTP, resulting in a single hit with 100% identity and coverage (AWF58599.1). This sequence belonged to the clinical *P. aeruginosa* strain AR_0443 (CP029147.1), which is reported to be resistant to imipenem (32 mg/L), meropenem (>8 mg/L), and doripenem (>8 mg/L). A resistome analysis of its complete genome found no genes encoding known carbapenemases.

The *mexT* gene, encoding for a regulator of the MexE-MexF-OprN multidrug efflux system of *P. aeruginosa*, was found to possess an 8-bp (GGCCAGCC) deletion (Figure 3), which leads to a frameshift in the coding sequence.

### 3.4. Expression Analysis of oprD Gene

The performed RT-qPCR analysis showed that the expression levels of the *oprD* genes in Pae51 and Pae52 were diminished compared to the carbapenem-susceptible *P. aeruginosa* PAO1 strain used as a calibrator (relative expression: 0.21 and 0.28, respectively).

### 3.5. WGS-Based Virulome Analysis

**Figure 4:**
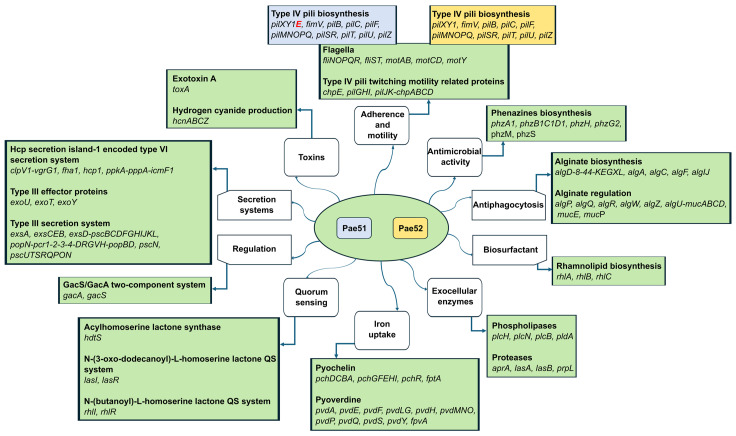
Virulome analysis of two *bla*_VEB-9_-positive *P. aeruginosa* isolates obtained from critically ill patients admitted to the University Hospital “St. Ivan Rilski”, Bulgaria.

Both isolates harbored a wide array of virulence-related genes encoding proteins involved in adherence, antimicrobial activity, antiphagocytosis, quorum sensing, iron uptake, secretion, the production of biosurfactant, and extracellular enzymes. Their complete list is given in Figure 4. All identified virulence-related genes are presented and grouped according to the classes defined in the Virulence Factor Database (VFDB) [38]. Also, the operon structures given are according to the Pseudomonas Genome DB [41].

Several virulence factors associated with type IV pili (*fimT*, *fimU*, *pilA*, *pilC*, *pilV*, *pilW*, and *pilY2*) and the biosynthesis of phenazines (*phzA2*, *phzB2*, *phzC2*, *phzD2*, *phzE1*, *phzE2*, *phzF1*, *phzF2*, and *phzG1*) were absent in Pae51 and Pae52 compared to the reference strain *P. aeruginosa* PAO1. The only difference between Pae51 and Pae52 was the presence of *pilE* in the genome of the former isolate, as illustrated in Figure 4.

The genomes of Pae51 and Pae52 contained genes encoding the type III secretion system effector proteins ExoT and ExoY. Additionally, they also harbored *exoU*, unlike the *P. aeruginosa* PAO1 strain, which possesses an *exoS*^+^/*exoU^–^* genotype. Many T3SS genes (listed in Figure 4) were also detected, indicating a functional T3SS machinery.

### 3.6. Phylogenomic Analysis of bla_VEB-9_-positive P. aeruginosa Strains

**Figure 5:**
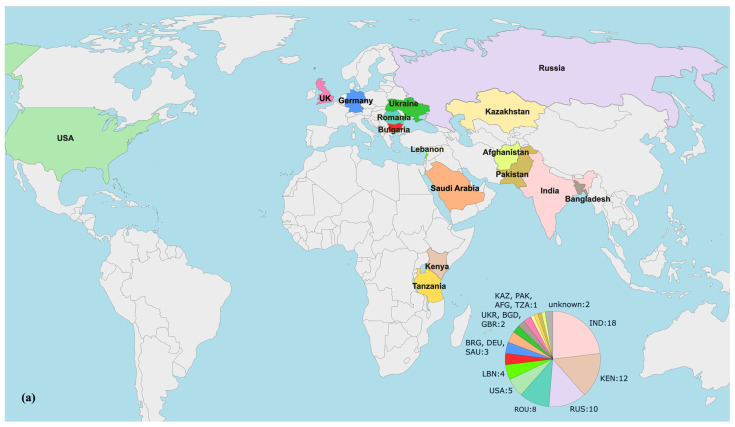

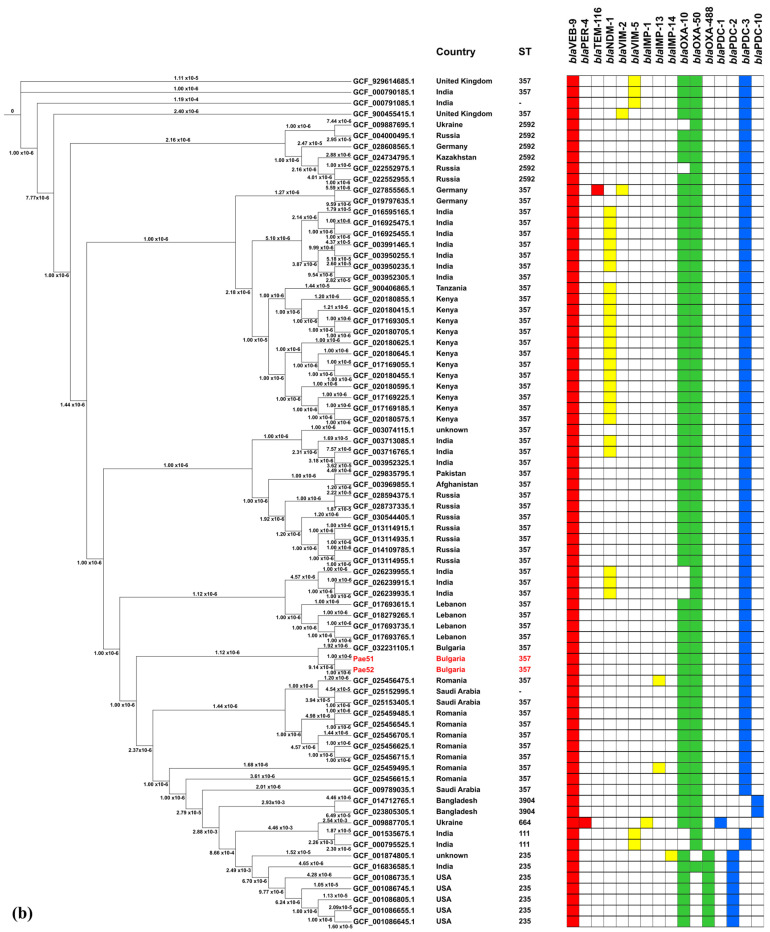
Phylogenomic analysis of *bla*_VEB-9_-positive *P. aeruginosa* strains with available genomes in the NCBI Nucleotide and The Pseudomonas Genome Database. (**a**) geographical origins of the isolates included. Countries displayed in the diagram are labeled with their respective three−letter ISO codes, as specified by the ISO 3166 international standard; (**b**) phylogenomic tree constructed by calling SNPs from core gene alignment of 78 *bla*_VEB-9_-positive isolates from Africa, Asia, Europe, and North America. A presence/absence matrix of β-lactamase-encoding genes is also given.

To examine the epidemiology of the investigated isolates, a set of 78 nonduplicate *bla*_VEB-9_-Positive *P. aeruginosa* genomes were collected as previously described. The geographic origins of these strains are given in Figure 5a. Next, a phylogenetic tree was constructed based on sequence variants in the core gene alignment, as depicted above. The result is shown in Figure 5b.

Our isolates, designated Pae51 and Pae52, were notably similar, differing by only four SNPs in their core genome regions. The genome most closely related to them was GCF_032231105.1, which belonged to a clinical *P. aeruginosa* Pae3125 strain from Varna (Bulgaria). This strain has been subjected to WGS, but our careful search found no published results on its genome in the literature.

As illustrated in the figure, 74.4% (58/78) of the *bla*_VEB-9_-containing isolates subjected to phylogenomic analysis belonged to the high-risk ST357 and were ubiquitously distributed across Africa, Asia, and Europe. The remaining isolates with a determined MLST profile were assigned to ST111 (2/78), ST235 (6/78), ST664 (1/78), ST2592 (6/78), and ST3904 (2/78).

All β-lactamase-encoding genes identified in the *bla*_VEB-9_-positive genomes included in the phylogenomic analysis are also listed in Figure 5b. The genetic determinants encoding OXA-10, OXA-50, and PDC-3 exhibit the highest levels of co-occurrence with *bla*_VEB-9_, at 91%, 91%, and 87.2% of the isolates, respectively. It is also noteworthy that 24 of the 78 isolates analyzed (30.8%) were found to be *bla*_NDM-1_-positive.

## 4. Discussion

*P. aeruginosa* is a common cause of HAIs, particularly in ICUs and surgical wards, which manifest as pneumonia, surgical site infections, urinary tract infections, and bacteremia [1]. According to the ECDC Annual Epidemiological Report 2020, 11,124 (12.7%) of patients staying in an ICU for more than two days developed at least one ICU-acquired HAI, and *P. aeruginosa* was the most frequently identified pathogen in ICU-acquired pneumonia (21.1%) and third, after *Escherichia coli* and *Enterococcus* spp., in urinary tract infections (14.3%). Furthermore, the incidence of CRPA isolates in these hospital wards in the European Union/European Economic Area (EU/EEA) countries was estimated at 26% [47].

Recent data on AMR show drastic differences in the prevalence of both nosocomial CRPA and MDR-PA isolates within the WHO EU/EEA Region. These problematic isolates tend to increase following the axes “west–east” and “north–south”, highlighting the Balkans, of which Bulgaria is a part, as a high-priority area [48]. In 2021, among the reported invasive *P. aeruginosa* isolates in Bulgaria, of which 54.2% were obtained from ICU patients, 32.5% and 31.3% were CRPA and MDR-PA, respectively [49].

The clinical *P. aeruginosa* isolates described in our investigation were problematic ICU pathogens. Antimicrobial susceptibility testing revealed retained activity of only two of the antibiotics studied: COL and CFDC. The clinical use of COL, an old polymyxin antibiotic available since the 1950s, has recently resurfaced as salvage therapy for severe Gram-negative infections, due to superbugs, including XDR-PA, which are resistant to all other antimicrobial agents. However, its therapeutic application should be limited both due to toxicity and low serum and tissue concentrations [50,51]. Despite recent approvals of novel antibiotics, there remain relatively few active treatment options for CRPA infections. Choices focus on the β-lactam–β-lactamase inhibitor (BL–BLI) combinations, such as CTT, CZA, and IMR, as well as the siderophore cephalosporin, CFDC [52]. Several studies have shown that MEV has activity against some clinical CRPA and MDR-PA strains [53,54]. Nevertheless, MEV, unlike the other new approved antibiotics, is not included as an option for DTR-PA infections in guidelines from the IDSA [55]. All the aforementioned BL–BLI combinations did not demonstrate in vitro activity against the two XDR-PA isolates subjected to the present study. Recently, Sid Ahmed et al. found that *bla*_VEB-9_ carriage in MDR-PA isolates from Qatar correlates with resistance to CZA and/or CTT. The authors reported that co-resistance to these antimicrobials is associated with the presence of *bla*_VEB-9_, *bla*_PDC-35_, *bla*_VIM-2_, *bla*_OXA-10_, and *bla*_OXA-488_, whilst CTT resistance alone is correlated with *bla*_VEB-9_, *bla*_PDC-35_, and *bla*_OXA-488_ [56]. AMR determinants encoding class A (*bla*_VEB-9_), class C (*bla*_PDC-3_), and class D (*bla*_OXA-10_ and *bla*_OXA-50_) β-lactamases were also identified in the *P. aeruginosa* Pae51 and Pae52 isolates. It is also worth mentioning the emergence of non-MBL-producing *Enterobacterales* demonstrating resistance to the new BL-BLI drugs. Recently Muresu et al. reported a *Klebsiella pneumoniae* isolate obtained from an Italian critically ill patient with COVID-19-related pneumonia demonstrating a remarkable resistance profile to several antimicrobial drugs, including CZA, MEV, and IMR. The authors found that the coexistence of both class A (KPC-31) and class D (OXA-181) carbapenemases, coupled with porin mutations, may be associated with the observed XDR profile [57].

Of the newly approved antibiotics, only CFDC demonstrated high in vitro activity (MIC = 0.5–1 mg/L) against the tested carbapenem-resistant and extensively drug-resistant *P. aeruginosa* isolates. It is a first-in-class catechol-substituted siderophore cephalosporin that has potent activity against a broad spectrum of aerobic Gram-negative pathogens, including carbapenem-resistant *Enterobacterales* and non-fermenting bacteria such as *P. aeruginosa*, *Acinetobacter baumannii*, and *Stenotrophomonas maltophilia* [58,59]. CFDC was approved by the U.S. Food and Drug Administration in late 2019 for the treatment of complicated urinary tract infections in adult patients, including pyelonephritis, and nosocomial pneumonia, including VAP, caused by susceptible aerobic Gram-negative bacteria [60]. Later, in 2020, CFDC was approved by the European Medicines Agency for complicated Gram-negative infections in adults with limited treatment options [61]. The efficacy and safety of CFDC have been shown in phase 2 and phase 3 clinical trials in patients with serious Gram-negative infections [62,63,64], but patients in these studies typically had other potential treatment options that allowed for randomization and trial enrollment. Thus, these trials provide limited information on the clinical effectiveness of the antibiotic for patients suffering from *P. aeruginosa* infections for whom no alternative treatment options exist. It is worth mentioning that, currently, CFDC has not yet been implemented in clinical practice in Bulgaria.

CFDC possesses the ability to overcome carbapenemases of different molecular classes and exhibit high stability against AmpC cephalosporinases, porin mutations, and efflux pumps complexly implicated in the carbapenem resistance of clinical *P. aeruginosa* strains [52,65]. Recent results from the SENTRY Antimicrobial Surveillance Program are very encouraging. These showed that 99.6% of the *P. aeruginosa* isolates tested and obtained in 2020 from the USA and Europe were susceptible to CFDC when interpreted according to the Clinical and Laboratory Standards Institute criteria. The antibiotic activity was also excellent against XDR-PA (97.3%), which showed lower susceptibility to the new BL–BLI combinations (IMR, 73%, CZA, 73.4%, and CTT, 72.3%). CFDC retained complete susceptibility in IMR-resistant isolates and remarkable susceptibility in CZA- and CTT-resistant isolates (91.6% and 88.3%, respectively), as well as with combined resistance to the three new antimicrobials (100%) [66]. A systematic review and meta-analysis performed by Karakonstantis et al., based on 78 studies, 87% of which were published between 2020 and 2023, showed excellent in vitro activity of CFDC against clinical isolates of *P. aeruginosa*. According to the presented results, the overall prevalence of CFDC-non-susceptible *P. aeruginosa* isolates was 1.4% [95% CI 0.5–4.0%], and among CRPAs, it was 3.5% [95% CI 1.6–7.8%] [67]. Data on the in vitro CFDC susceptibility of clinical CRPAs in Bulgaria are scarce and concern only single-MBL-producing isolates. A recent study found 100% susceptibility to CFDC in the testing of eight New Delhi metallo-β-lactamase-1 (NDM-1)-producing *P. aeruginosa* isolates from a tertiary hospital in Sofia [68]. In contrast, Stoykov et al. reported three *P. aeruginosa* isolates also from Sofia producing IMP-type MBLs, two of which showed resistance to CFDC (MIC values of 4–8 mg/L) [69].

The widespread use of β-lactams and the adaptive capacity of the *P. aeruginosa* genome have led to a significant increase in the antimicrobial resistance of the pathogen over the past decade. Acquired resistance to these antibiotics is associated with both enzymatic and non-enzymatic mechanisms. Enzymatic mechanisms include the production of various types of ESBLs, carbapenem-hydrolyzing enzymes, mainly of class B (MBLs) and less frequently of class A, and the hyperproduction of the chromosomal AmpC β-lactamase of class C [70,71]. Despite the phenotypically manifested carbapenem resistance of the Pae51 and Pae52 isolates detailed in this study, WGS-based resistome analysis did not identify any AMR determinants for either class B- or class A-acquired carbapenemases. The only identified genes associated with β-bactam resistance were *bla*_VEB-9_, which encodes the VEB-9 ESBL of class A, *bla*_PDC-3_ (class C intrinsic *Pseudomonas*-Derived Cephalosporinase-1), *bla*_OXA-10_, and *bla*_OXA-50_ (both class D β-lactamases).

The subject of this study, VEB-9 (formerly known as VEB-1a) ESBL, was initially detected in two clinical *P. aeruginosa* isolates from ICU patients in Kuwait that demonstrated resistance to CAZ and cefotaxime, as well as a synergistic effect between CAZ and clavulanic acid [72]. As a representative of molecular subclass A2 and functional group 2be, VEB-9 is responsible for the resistance of the studied Pae51 and Pae52 isolates to ureidopenicillins, extended-spectrum cephalosporins, including CAZ and FEP, and aztreonam [27,73]. The only reported variant of VEB-type ESBLs among MDR-PA in Bulgaria is VEB-1, and previously identified nosocomial *bla*_VEB-1_-positive *P. aeruginosa* isolates have been shown to belong to two international high-risk clones, ST111 and ST244 [74,75,76]. Over the last decade, VEB-9-producing *P. aeruginosa* isolates have been found in Thailand, Poland, and Qatar [56,77,78,79]. For the first time in Europe, Laudy et al. detected *bla*_VEB-9_-positive *P. aeruginosa* (7.7%) among CAZ-resistant and/or FEP-resistant clinical isolates from four hospitals in Warsaw, Poland, between 2000 and 2014. The authors established the clonal relatedness of the isolates using PFGE typing. All VEB-9 producers were assigned to one pulsotype and two subtypes, PT M and PT Ma/Mb, respectively [78]. Later, Sid Ahmed et al. conducted the WGS of 75 selected MDR-PA clinical isolates collected from several hospitals in Qatar in 2014–2015 to analyze the dominant STs and AMR determinants of β-lactam resistance. VEB-9 was reported to be the most prevalent ESBL (25.3%), and 18 of the 19 *bla*_VEB-9_-containing *P. aeruginosa* isolates were part of the high-risk clones ST235 (*n* = 8), ST357 (*n* = 7), and ST308 (*n* = 3) [79]. As presented above, our VEB-9 producers were also classified as ST357.

The phylogenomic analysis revealed that ¾ of all *bla*_VEB-9_-positive *P. aeruginosa* genomes worldwide also belong to ST357. Alarmingly, 41.3% of these, sourced from India, Kenya, and Tanzania, also carry the *bla*_NDM-1_ gene encoding the NDM-1 MBL. The combination of VEB-9 and NDM-1 confers resistance to all β-lactams, including those combined with β-lactamase inhibitors, significantly restricting the available treatment options [80].

The other sequence types were represented by single *bla*_VEB-9_-positive strains involved in the phylogenomic analysis. However, ST2592 (*n* = 6) warrants attention due to its presence across a broad geographic region, including Russia, Ukraine, Germany, and Kazakhstan. The ongoing military conflict in that area could potentially facilitate the rapid spread of VEB-9-producing ST2592 *P. aeruginosa* isolates. Therefore, increased stewardship practices in the surrounding countries may be necessary to manage this risk.

The *bla*_VEB-9_ gene in Pae51 and Pae52 was found to be part of a complex, class 1-like integron located on the chromosome. Despite its location, the presence of numerous genes encoding proteins necessary for the transfer of mobile genetic elements, situated nearby, underscores the potential for horizontal gene transfer to other isolates. Extensive comparison with annotated prokaryotic transposons showed components of Tn*3*-family transposons Tn*5086*-CP054343 (found in *E. coli* SCU-164) and Tn*1412*-L36547 (described in *P. aeruginosa* 2293E), suggesting complex genetic transpositions and rearrangements in the VEB-9 region. A BLASTN search using the *bla*_VEB-9_ gene and its surrounding regions from our isolates against the Nucleotide collection (nr/nt) database revealed 14 hits in complete *P. aeruginosa* genomes, each with query coverage of 98% or higher. This suggests that the organization of the *bla*_VEB-9_ context in the Pae51 and Pae52 isolates is common for the species.

In Gram-negative pathogens, carbapenem hydrolysis seems not to be restricted to the main carbapenem-hydrolyzing class D β-lactamase (CHDL) groups, including OXA-23, OXA-24/40, OXA-48, and OXA-51 found in *Acinetobacter* species. The OXA-10 β-lactamase of class D identified in Pae51 and Pae52 may also possess weak carbapenemase activity at a level that is the same as or higher than that of, e.g., OXA-58 [81]. There are opposing claims in the literature about the nature of the other class D β-lactamase detected, OXA-50, and its derivatives. Some authors present them as naturally occurring in all *P. aeruginosa* strains [82,83], while others refer them to acquired oxacillinases with carbapenemase activity [84]. A few years ago, Petrova et al. reported three OXA-50-producing CRPA isolates from a university hospital in Plovdiv, Bulgaria, which lacked AMR determinants for class B and A carbapenemases but demonstrated weak carbapenemase activity when performing the modified Hodge test [85,86]. The authors recognized a secondary role for OXA-50 in carbapenem resistance, and overexpression of the MexXY-OprM efflux pump combined with OprD deficiency and/or overexpression of the MexAB-OprM pump were identified as the primary mechanisms [84].

Our analysis of the *oprD* coding sequence revealed numerous missense mutations and a frameshift mutation, the majority of which impact the loop regions of the encoded porin. Similar observations have been previously reported in CRPA isolates, indicating that alterations in OprD reduce its ability to bind imipenem, thereby conferring resistance to this antibiotic [87]. Additionally, the subsequent expression analysis established reduced *oprD* expression, which also contributes to the observed imipenem resistance in the Pae51 and Pae52 isolates. It was accompanied by an 8-bp deletion in the *mexT* gene, which resulted in a frameshift. This variant was present in all 29 MDR *P. aeruginosa* clinical isolates collected from various laboratories in Kerala, India, between 2012 and 2016 in an earlier study [87], and *oprD* was downregulated in 89.7% (*n* = 26) of these isolates. It is worth noting that, unlike most Resistance Nodulation Division-type efflux systems in *P. aeruginosa*, MexT serves as a positive regulator of MexEF-OprN expression. This observation supports a previous hypothesis that in strains with wild-type and/or inactivated *mexT,* mutations in other, yet unidentified genes may be crucial for the hyperexpression of *mexEF-oprN* and the reduced production of OprD [88].

Consistent with previous studies showing an association of ST357 with the *exoU*^+^/*exoS^−^* T3SS genotype [20,21], our virolome analysis revealed that Pae51 and Pae52 (ST357) possess this specific genotype. ExoS and ExoU T3SS effector proteins play an essential role in infectious pathogenesis. ExoS leads to delayed apoptotic cell death, whereas ExoU is associated with a highly cytotoxic phenotype of *P. aeruginosa* and induces rapid host–cell lysis [22]. ExoU is considered the major T3SS cytotoxin because it has the greatest impact on disease severity, being associated with severe acute lung injury, sepsis, and mortality [89,90,91]. It is recognized as an independent risk factor for early mortality related to *P. aeruginosa* bloodstream infections [91]. Furthermore, the secretion of the ExoU cytotoxin is considered a marker for highly virulent *P. aeruginosa* strains obtained from patients with hospital-acquired pneumonia (HAP) [92], such as the Pae51 and Pae52 isolates we studied. Different investigations have shown that *exoS* and *exoU* are almost always mutually exclusive in the genome of *P. aeruginosa* [21,89,93,94,95], which was also found in the present study. This finding may be related to the possibility that the expression of each of these genes provides increased fitness in different ecological niches [96]. In contrast to these results, there are reports of the simultaneous existence of *exoS* and *exoU* in a significant number of clinical *P. aeruginosa* isolates [97,98,99,100,101,102].

As mentioned, the O-specific antigen in *P. aeruginosa* lipopolysaccharide was determined via in silico serotyping. The analysis showed that both isolates studied belong to the O11 serotype. Regarding serotype, our finding is consistent with the existing literature, which demonstrates a strong association between O11 and the MDR phenotype [103]. The O11 serotype is usually linked to the *exoU*-positive genotype as well as to the high-risk clone ST357 [21], as was also found in our study. Interestingly, the O11 serotype is associated with a worse prognosis in HAP/VAP and bloodstream infections among critically ill patients [103,104].

## 5. Conclusions

To the best of our knowledge, this is the first report of *bla*_VEB-9_-positive XDR-PA isolates in Bulgaria presenting detailed genomic and phylogenomic analyses. Pae51 and Pae52 belonged to high-risk clone ST357, as well as to the O11 serotype and *exoU*-positive genotype, both associated with a worse prognosis for HAI outcomes in critically ill patients. Moreover, this study provides extensive information on the worldwide distribution of VEB-9-producing *P. aeruginosa* isolates combined with their MLST and β-lactamase-encoding gene profiles. Genetic determinants encoding OXA-10, OXA-50, and PDC-3 β-lactamases showed the highest levels of co-occurrence with *bla*_VEB-9_, and most isolates (74.4%) were assigned to high-risk clone ST357.

HAIs caused by therapeutically problematic and highly virulent *P. aeruginosa* pose a growing threat to public health. Therefore, the development of novel antimicrobial strategies for such pathogens should be an essential part of infection control stewardship practices in hospitals, especially in ICU wards.

## 6. Limitations of the Study

The number of the included *bla*_VEB-9_ *P. aeruginosa* isolates obtained from the aforementioned university hospital was low. The main purpose of the present study was to explore the genomic characteristics (resistance and virulence determinants) of two VEB-9-producing XDR-PA isolates in comparison to all *bla*_VEB-9_-positive *P. aeruginosa* strains with available genomes originating from all over the world. This study does not provide information regarding the prevalence and antimicrobial resistance of VEB-9-producing *P. aeruginosa* isolates in either a specific hospital or the country. The expression of *mexEF-oprN* was not measured.

## Figures and Tables

**Table 1:** Antimicrobial susceptibility testing of studied VEB-9-producing *P. aeruginosa* isolates.

Antimicrobial Agents	MIC [mg/L] and Interpretation	
	Pae51	Pae52
Piperacillin	>256 R	>256 R
Piperacillin-tazobactam ^a^	>256 R	>256 R
Ceftazidime	>256 R	>256 R
Ceftazidime-Avibactam ^a^	>32 R	>32 R
Cefepime	>256 R	>256 R
Ceftolozane-Tazobactam ^a^	>8 R	>8 R
Cefiderocol	1 S	0.5 S
Imipenem	>32 R	>32 R
Imipenem-Relebactam ^a^	>16 R	>16 R
Meropenem	>32 R	>32 R
Meropenem-Vaborbactam ^b^	>16 R	>16 R
Aztreonam	64 R	64 R
Amikacin	32 R	48 R
Tobramycin	16 R	16 R
Ciprofloxacin	>32 R	>32 R
Levofloxacin	>32 R	>32 R
Colistin	1 S	1 S

MIC, minimum inhibitory concentration; R, resistant; S, susceptible. ^a^ The concentration of tazobactam/avibactam/relebactam was fixed at 4 mg/L. ^b^ The concentration of vaborbactam was fixed at 8 mg/L.

**Table 2:** Whole genome-based characterization of studied VEB-9-producing *P. aeruginosa* isolates.

Isolate	Genome Size (Mb)	GC%	N50 (bp)	Number of Contigs	Serotype	ST	Alleles						
							*acsA*	*aroE*	*guaA*	*mutL*	*nuoD*	*ppsA*	*trpE*
Pae51	6.72	66.04	425,538	41	O11	357	2	4	5	3	1	6	11
Pae52	6.72	66.03	395,429	47	O11	357	2	4	5	3	1	6	11

## Data Availability

The whole-genome shotgun sequencing projects of the *P. aeruginosa* Pae51 and Pae52 isolates were deposited in GenBank under BioProject accession number PRJNA1138419 (assemblies JBFQFS000000000 and JBFQFR000000000, respectively).
